# Does negative auto-regulation increase gene duplicability?

**DOI:** 10.1186/1471-2148-9-193

**Published:** 2009-08-07

**Authors:** Tobias Warnecke, Guang-Zhong Wang, Martin J Lercher, Laurence D Hurst

**Affiliations:** 1 Department of Biology & Biochemistry, University of Bath, UK; 2 Bioinformatics Group, Heinrich-Heine-University Düsseldorf, Germany

## Abstract

**Background:**

A prerequisite for a duplication to spread through and persist in a given population is retaining expression of both gene copies. Yet changing a gene's dosage is frequently detrimental to fitness. Consequently, dosage-sensitive genes are less likely to duplicate.

However, in cases where the level of gene product is controlled, via negative feedback, by its own abundance, an increase in gene copy number can in principle be decoupled from an increase in protein while both copies remain expressed. Using data from the transcriptional networks of *E. coli *and *S. cerevisiae*, we test the hypothesis that genes under negative auto-regulation show enhanced duplicability.

**Results:**

Controlling for several known correlates of duplicability, we find no statistically significant support in either *E. coli *or *S. cerevisiae *that transcription factors under negative auto-regulation hold a duplicability advantage over transcription factors with no auto-regulation.

**Conclusion:**

Based on the analysis of transcriptional networks in *E. coli *and *S. cerevisiae*, there is no evidence that negative auto-regulation has contributed, on a genome-wide scale, to the variability in gene family sizes in these species.

## Background

Increasing a gene's dosage can have very different effects. Occasionally, producing more of the same can confer a selective advantage. For example, high copy numbers of the *gch1 *gene are linked to resistance against antifolate drugs in the malaria parasite *Plasmodium falciparum*. As individuals with elevated copy numbers are notably more frequent in populations where these drugs are in use, this suggests the action of natural selection [1]. On a genome-wide level, duplicated isozymes in yeast show high retention rates, presumably because increased dosage facilitates high enzymatic flux [2,3]. Frequently, however, radical alterations to a gene's dosage are deleterious. Sopko *et al*. (2006) systematically overexpressed individual genes in yeast and discovered that growth phenotypes were measurably reduced for more than 700 (~15%) of the genes tested. In some cases, the authors suggest, decreased fitness is likely owing to overexpression of genes that would normally be expressed only periodically, such as genes involved in the cell cycle [4]. Deleterious effects have also been attributed to relative rather than absolute excess in protein, a phenomenon known as dosage imbalance [5,6].

### Gene duplication and gene dosage

One process that can bring about instant, permanent, and sometimes drastic increases in gene dosage is gene duplication. As suggested by the example of *gch1 *above, this immediate, dosage-enhancing effect of duplication might be what allows the duplicated gene to persist in the population and eventually rise to fixation. In fact, some degree of expression initially after duplication seems a strong prerequisite for a new duplicate to escape pseudogenization. If a gene fails to be expressed, there is little leverage for selection to promote its retention.

Many genes, then, face conflicting requirements when it comes to duplicating successfully: the new copy stands little chance of rising to fixation and being retained long-term, perhaps through acquiring new or subdividing old functions, if it is not expressed. But being expressed alongside the old copy implies increased dosage, which is frequently deleterious. A possible genomic signature of this problem can be witnessed in yeast where genes involved in protein complexes, where changes in the expression of one gene will alter dosage balance, have fewer duplicates [5,7]. This is specifically the case for hetero-complexes (at least two different types of subunits) whilst homo-complexes, where relative dosage should *a priori *not be an issue, show duplicability comparable to monomers [8].

### Homeostatic genes: Hypothesis

While some genes, then, may be caught up in the above predicament, others might be uniquely placed to negotiate it, namely genes whose product level is controlled in a homeostatic fashion. "Homeostatic" we take to mean any situation where the abundance of a protein is regulated, in a negative feedback loop, by the abundance of that same protein. Critically, under this type of regulatory set-up an increase in the number of active production units (gene copies) does not inevitably lead to a net gain in product (protein). Thus, when homeostatically controlled genes duplicate, both copies can, theoretically, remain expressed without incurring any potential costs associated with altered dosage. Under this model, negative feedback removes a potential barrier to duplication rather than necessarily providing an instant selective advantage that might lead to fixation. However, this is not to say that instant benefits may not exist. Having two functional copies of the same gene can, for example, lead to reduced noise [9], a fitness benefit [10] that, interestingly, has also been attributed to negative feedback regulation [11]. In the longer term, a homeostatic set-up might allow for rapid functional divergence of one copy without affecting the function of the other. For example, if one copy evolved to be expressed in a tissue-specific fashion, this would not compromise the quantity of protein in other tissues.

Based on these considerations, it is worth asking whether, other things equal, genes under homeostatic control exhibit greater duplicability than comparable genes not regulated via negative feedback. Below, we test this hypothesis, using data from two well-studied microbial organisms.

### *E. coli *as a model system

One molecular system where negative feedback regulation is common is the transcription network of *E. coli *[12,13]. Of >150 transcription factors (TFs) with experimentally verified interactions, a large proportion (>50%) exhibit regulation via negative feedback.

There is further cause to suggest the *E. coli *transcription network may provide a suitable candidate system to explore the issue of dosage-related differential duplicability. First, negative feedback in this system is exclusively via auto-regulation, i.e. the TF directly represses its own transcription. In multi-layer feedback systems, on the other hand, multiple components have to be transcribed and translated successively before feedback can take effect. Such multi-layer systems should incur greater time lags in feedback, and hence a greater probability of protein production to overshoot target levels.

Second, several studies have previously identified TFs as typically having *low *duplicability in comparison to other gene ontology categories [4,14-16]. By contrast, Cosentino Lagomarsino *et al*. (2007) have noted that TFs with negative auto-regulation are duplicated at ordinary rates [17]. This is consistent with negative auto-regulation providing an escape route to duplication for TFs, which typically have a hard time duplicating successfully (outside of whole-genome duplications, see e.g. [18]).

Finally, we already know duplicability to co-vary with a number of gene attributes including a gene's dispensability [19], its level of connectedness in protein networks [15,16], and its biochemical function [15,18,20,21]. Therefore, simply comparing TFs regulated via negative feedback with the remainder of genes in the genome is unlikely to yield meaningful insights so that identifying an adequate control population must be a paramount concern. Transcriptional networks provide a natural internal control group, i.e. we can compare TFs with negative auto-regulation to TFs that are not controlled via negative feedback. This excludes protein connectedness and biochemical function as potential confounding factors. We thus decided to test the hypothesis that homeostatic regulation endows genes with enhanced duplicability by analysis of the *E. coli *transcription network.

### Yeast as a model system

In testing a general evolutionary hypothesis, it is of course optimal to analyze more than one model system. Next to *E. coli*, the best-characterised transcription network is that of the baker's yeast *Saccharomyces cerevisiae*. In contrast to *E. coli*, however, considerably fewer cases of auto-regulation can be found in yeast [22]. Thus, while below we report results for both *E. coli *and *S. cerevisiae*, any comparison of negatively auto-regulated to not auto-regulated TFs in yeast necessarily suffers from low statistical power.

## Results

### No evidence for higher duplicability of negative auto-regulators

To determine whether genes under homeostatic control exhibit enhanced duplicability, we assessed duplication patterns for genes in the transcription network of *E. coli*, where a substantial proportion of TFs (>50%) show negative auto-regulation [12,13]. Our final dataset (see Methods) contains 155 TFs of which 62 are negative auto-regulators. As the number of TFs with both positive and negative auto-regulation ("dual") is limited (N = 9), we focus on TFs with either positive or negative auto-regulation.

We employ the proportion of single-gene families amongst all gene families represented in the respective regulatory class (see Table 1) as our preferred measure of duplicability. This binary distinction between single- and multi-gene families has been widely used to characterize differential duplicability between genes grouped according to a feature of interest [7,8,15,19]. As this measure is based on surveying gene families in the extant *E. coli *genome, it does not directly chart duplication dynamics but rather amalgamates possibly quite different gain and loss histories of individual gene families. However, for genomes at equilibrium with regard to duplication activity this simple compound index should provide a good approximation for long-term duplicability trends, particularly across larger groups of genes, which is what we are dealing with. A more gene-centred way of assessing duplicability would be to trace individual gain/loss events across an informative phylogeny. While it would certainly be desirable to directly estimate duplication rates in this manner, this approach comes with substantial added complexity. For example, confident reconstruction of duplication events on bacterial phylogenies is difficult in the presence of ubiquitous lateral gene transfer (LGT, see below). Furthermore, it is difficult to estimate what phylogenetic depth is required to both yield representative trends across groups of genes and at the same time not compromise the assumption that regulatory interactions are relatively stable. We thus confine our analysis to the simple yet informative gene group-centred measure of duplicability.

**Table 1:** Duplicability in the transcriptional networks of *E. coli *and *S. cerevisiae*

	Negative auto-regulation	Positive auto-regulation	Dual auto-regulation	No auto-regulation
*E. coli*				

Family size = 1	10	4	2	14

Family size >1	52 (25)	20 (10)	7 (7)	46 (22)

*d*	0.71	0.71	0.78	0.61

*S. cerevisiae*				

Family size = 1	4	5	0	86

Family size >1	0	5	0	26

*d*	0	0.5	0	0.23

The number of transcription factors belonging to gene families of Family size = 1 (unduplicated) or Family size>1 (duplicated) are given by species and type of auto-regulatory interaction. The number of independent gene families contributing members to the sample is given in parentheses. Duplicability (*d*) for each regulatory class was determined as *d *= 1-N(F = 1)/(N(F = 1)+N(F>1)) based on the number of independent families (see Lin *et al*. 2007). Note that estimates of *d *can become misleading for low sample sizes so that results should be interpreted with care. No internal comparison is significant at p < 0.05 using Fisher's exact tests.

Comparing auto-regulators to TFs without auto-regulation we find no significant difference in duplicability (Table 1, negative auto-regulators: Fisher's exact test P = 0.45; positive auto-regulators: P = 0.74). When we limit our analysis to genes for which we can rule out, with reasonable confidence, that they entered the *E. coli *genome via LGT, we recover very similar results (Table 2).

**Table 2:** Duplicability in the transcriptional network of *E. coli *(excluding LGT candidates)

	Negative auto-regulation	Positive auto-regulation	Dual auto-regulation	No auto-regulation
Family size = 1	7	2	0	9

Family size >1	27 (16)	6 (3)	2 (2)	21 (15)

*d*	0.69	0.6	1	0.63

The number of transcription factors belonging to gene families of size Family size = 1 (unduplicated) or Family size>1 (duplicated) are given by type of auto-regulatory interaction. This table corresponds to Table 1 but transcription factors with prior evidence for lateral gene transfer (see Methods) have been excluded. No internal comparison is significant at p < 0.05 using Fisher's exact tests.

TFs with and without auto-regulation might differ systematically with regard to known correlates of duplicability, obscuring a potential contribution of regulatory type to differential duplicability. We investigated several potential confounding factors.

### Regulatory types do not differ in gene complexity

**Figure 1:**
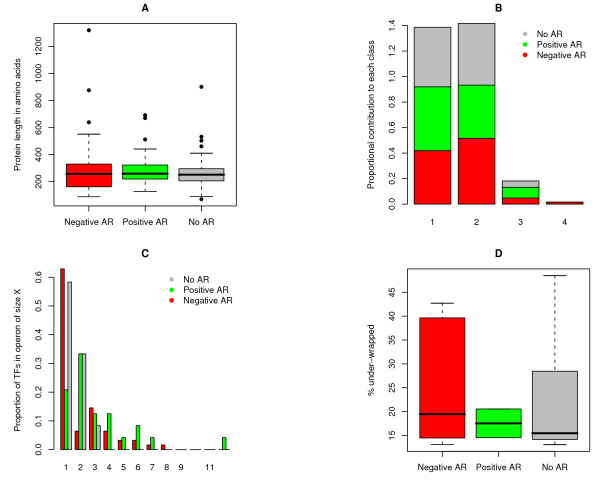
**Known correlates of duplicability have no confounding effect**. *E. coli *transcription factors under negative, positive or no auto-regulation are compared with regard to (A) protein length, (B) the number of different protein domains, (C) the number of genes present in the operon which they form part of, and (D) the proportion of under-wrapped residues, all of which are known correlates of duplicability. There are no significant differences between negative auto-regulators and transcription factors without auto-regulation. The only significant difference between regulatory types is an enrichment of positive auto-regulators in polycistronic (operon size >1) transcripts (P = 0.0002). See Table 1 and main text for sample sizes. AR: auto-regulation.

He and Zhang reported for yeast that genes retained after duplication had, on average, longer protein sequences, a greater number of functional domains and more *cis*-regulatory elements [23]. The authors suggest that this may be because these genes provide greater scope for sub- and subsequent neo-functionalization, so that selection would have favoured their retention over less complex genes. This argument is based on general evolutionary dynamics rather than features specific to yeast, so that similar biases may apply to *E. coli*. While we find no difference between duplicated and non-duplicated genes across our limited TF sample (Mann-Whitney U P = 0.08), more importantly, there are no significant differences in protein length (all pairwise MWU P > 0.05) or in the number of functional domains (all pairwise MWUs P > 0.05; ignoring domain repeats) between TFs of different regulatory type (Figure 1A&1B). As the majority of genes in *E. coli *are expressed as part of polycistronic transcripts, gene-specific comparisons of *cis*-regulatory elements is not possible.

### Negative auto-regulators and TFs without auto-regulation do not differ in operon structure

The fact that *E. coli *genes are frequently organized into multi-gene operons might in and of itself be a confounding factor. In *C. elegans*, genes in operons exhibit reduced duplicability compared to monocistronic genes [24,25]. The reasons for this remain essentially unresolved. However, part of the explanation might be that (inverted) tandem duplications of a gene inside an operon can disrupt the structure of that operon [24], a mutational bias that would also apply to bacteria.

As a precautionary measure, we thus decided to test whether there are systematic differences in operon membership between auto-regulators and non-auto-regulators. Consistent with results from *C. elegans*, duplicated TFs are enriched in monocistronic transcription units (55.2% v 46.7%), albeit not significantly so (P = 0.34). Further, there are no differences between negative auto-regulators and TFs without auto-regulation (P = 0.48). Duplicated positive auto-regulators on the other hand are significantly more likely to be part of polycistronic transcripts (P = 0.0002; Figure 1C). Why this is the case is currently unclear. However, this should reduce duplicability of positive auto-regulators whereas we find it to be nominally higher (Table 1).

### Regulatory types do not differ in under-wrapping

Finally, we explored whether genes from different regulatory types vary systematically in relation to under-wrapping. Under-wrapping quantifies the extent to which hydrogen bonds at the protein backbone are accessible to water and thus susceptible to hydration, which can jeopardize the structural integrity of the protein. The degree of under-wrapping has been argued to serve as a proxy for how reliant a protein is on binding partnerships to maintain its structural integrity ([26] and references therein). In line with the dosage balance hypothesis, Liang and colleagues recently found highly under-wrapped proteins to be, on average, less duplicable in *E. coli *as well as several eukaryotic genomes [26]. This need not necessarily hold true for individual genes. It has also been suggested that poorly packed, disordered proteins may exhibit high conformational versatility and hence evolvability [27]. In as far as under-wrapping captures such conformational flexibility, highly under-wrapped proteins may therefore exhibit high duplicability in cases where duplicate retention is promoted through sub-/neofunctionalization rather than dosage concerns. However, the results of Liang *et al*. strongly suggest that, on a genome-wide level, the effect of under-wrapping on duplicability is predominantly negative.

This is certainly evident for the TFs investigated here. Across regulatory types, TFs of multi-gene families show markedly reduced under-wrapping compared to singletons (median proportion of bonds under-wrapped in duplicated genes: 15.84%; non-duplicated genes: 28.46%, MWU P = 0.006). Higher under-wrapping for singleton genes can also be observed within regulatory categories (No auto-regulation: median proportion of bonds under-wrapped in duplicated genes: 14.51%; non-duplicated genes: 28.46%, P = 0.042; negative auto-regulation: duplicated genes: 18.85%; non-duplicated genes: 33.58%, P = 0.098; positive auto-regulators were not analyzed further as under-wrapping information was available for only two genes). However, we find no significant differences in under-wrapping characteristics between regulatory types (Figure 1D), suggesting that differences in under-wrapping do not bias our assessment of duplicability.

### No evidence for higher duplicability of negative auto-regulators in yeast

To corroborate our findings, we repeated our analysis for gene duplications in the transcription network of the yeast *S. cerevisiae*. In contrast to *E. coli*, only 15% of yeast transcription factors (14 out of 112) are auto-regulatory [22]. As evident from Table 1, genes with negative auto-regulation show no evidence of higher duplicability (Fisher's exact test P = 0.57). However, as sample size is severely limited, only extreme differences in duplicability would lead to a significant result in our data set. Due to the small sample size, we also refrained from an analysis of potential confounding factors.

## Discussion

We have argued that, *a priori*, genes under homeostatic control might be expected to show greater duplicability than genes without negative feedback regulation. This is principally because negative feedback can decouple an increase in functional production units (i.e., duplication of actively transcribed genes) from an increase in product (which can be detrimental to the organism because it disrupts absolute or relative dosage requirements). However, we find little support for this hypothesis, neither in the transcription network of *E. coli*, a system where negative feedback regulation is common, nor in the transcription network of *S. cerevisiae*: TFs under negative auto-regulation do not differ significantly from TFs without auto-regulation in terms of their duplicability. We note that, in line with our hypothesis, duplicability is at least nominally higher for negative auto-regulators in *E. coli *(d = 0.71 *vs*. d = 0.61 in TFs without auto-regulation; see Table 1). Surprisingly, the same is true for positive auto-regulators; however, genes subject to positive feedback control might from the outset be considered unlikely candidates to suffer from increased dosage.

The above analysis suggests that there is no duplicability bias favouring negative auto-regulators. Several factors might contribute to the absence of such an effect. Notably, only genes for which dosage distortions would have negative fitness consequences may enhance their duplicability through negative auto-regulation. The findings of Sopko and colleagues [4] suggest that only a moderate proportion of genes may fall into this category. Inspecting a sample of genes without prior information on whether dosage alterations brought about by duplication would indeed reduce fitness, then, we probably ought to assume that only a minority stand to benefit from auto-regulation in the first place. Furthermore, our model presupposes that negative feedback regulation leads to post-duplication dosage below what is expected from the doubling of gene copy number. However, negative feedback control is never instantaneous, so that protein levels after initiation of transcription can temporarily overshoot pre-duplication levels. Some genes might cope well with transiently elevated protein levels, but for others the failure to reign in protein levels even for a short period might be detrimental, further reducing the pool of genes that fit our original model.

Alternatively, a duplicability bias may exist, yet we might fail to detect it. Given the limited number of genes that might stand to benefit from reigning in dosage, across all genes, any signature of differential duplicability might be subtle. Further, in *E. coli*, we observed tendencies towards greater under-wrapping, as well as a significant enrichment in multi-gene operons, in both negative and positive auto-regulators (Figure 1D&1C). These covariances might further weaken an already weak signal, as they should impede rather than facilitate duplicability.

### Are the model systems adequate?

The *E. coli *transcription network offers some distinct advantages as a model to investigate duplicability (auto-repression; large number of negative auto-regulation interactions; natural control group), but it also comes with some severe caveats. For example, our analysis assumes that regulatory interactions in the extant *E. coli *genome reflect interactions at the time of duplication. While Lagomarsino *et al*. (2007) found significant similarity in auto-regulation within TF families [17], this has recently been suggested to largely result from convergent evolution rather than conservation of ancestral regulation [28]. A further notable complication arises from the observation that family expansions are, to substantial parts, owing to LGT [28,29]. Dosage-related concerns may also influence the fate of LGT-derived paralogs, but whether or not this is a relevant issue will critically depend on how similar the transferred gene is to the resident gene at both the sequence and the regulatory level. Although our results hold when we exclude TFs derived from LGT, sample sizes become rather small; thus, we are unlikely to detect any effect even if there was one.

Our failure to find a statistically significant difference between TFs with and without auto-regulation in *S. cerevisiae *may equally be owing to small sample size as the yeast transcription network provides comparatively few examples of negative auto-regulation [22]. This is not to say that, sample size issues aside, genomic analysis of duplicability in yeast does not have its own pitfalls. Most notably, *S. cerevisiae *[30], like many other genomes, is the product of a whole-genome duplication (WGD). Our hypothesis of a duplicability advantage for negative auto-regulators, however, principally applies to smaller scale duplications. For WGD duplicates, the reverse may be true. Notably, genes involved in protein complexes have been preferentially retained following WGD in *Paramecium tetraurelia *[31], and this has been interpreted as selection on dosage balance: Genes involved in a single protein complex experience proportional dosage increases, and individual genes should thus be less likely to be lost – they are stuck at the new dosage. Under this scenario, genes with negative auto-regulation do not participate in the general dosage increase, and might thus not be preferentially retained after a WGD event.

### Alternative model systems

The above section highlights that the genome-level impact, if any, of negative feedback on gene family evolution will strongly depend on the specific genome under consideration. Further, where radical genomic transitions such as WGDs are implicated, current genome composition may not be at equilibrium. As a corollary, extant patterns of duplicability need not be representative of the forces driving family size evolution in the longer term. For example, dosage sensitivity might provide a short-term retention bias but be less important in long-term family size evolution, as has been suggested for the genome of *P. tetraurelia *[32]. Thus, convincingly establishing a feedback-duplicability link in genomic data will be non-trivial and require intimate knowledge of the system under scrutiny.

Unfortunately, we know of no other systems equally or better suited to investigate such a link. More suitable model systems might be found in the transcription networks of higher eukaryotes; however, despite recent progress (*e.g*. [33]), our knowledge about these networks remains partial and biased towards developmental pathways.

Negative feedback loops are, of course, not restricted to transcriptional networks. Lareau *et al*. (2007) found that splice-regulatory proteins of the SR protein family in mammals affect their own splicing pattern to generate isoforms that are subject to nonsense-mediated decay (NMD), a negative auto-regulation circuit [34]. Yet making an argument for enhanced duplicability based on a single gene family would be anecdotal at best. Another candidate system comprises regulatory circuits involving microRNAs, which have been identified as frequently homeostatic in nature [35,36]. In as far as regulation by microRNAs serves as a marker for genes partially under homeostatic control, we would expect microRNA-regulated genes to enjoy some duplicability advantage. It is intriguing to note in this regard that mammalian duplicates have recently been reported to harbor more microRNA target sites and be regulated, on average, by more microRNA species than their singleton counterparts [37]. Evidently, this is by no means conclusive support for a dosage-related duplicability bias. Further, as repression mediated by microRNAs is typically weak [38], so that post-duplication dosage is likely to be above ancestral levels, duplicability biases might be rather subtle.

## Conclusion

We have argued that negative feedback regulation can decouple increases in gene copy number (via duplication) from an increase in the product of that gene and therefore equip genes with a duplicability advantage. We find no support for differential duplicability owing to regulatory set-up in the transcriptional networks of either *E. coli *or *S. cerevisiae*. While these model systems suffer from specific shortcomings that might affect conclusions, we suggest it best to suppose, in the absence of evidence to the contrary, that there is no link between negative auto-regulation and duplicability as far as shaping genome-wide differences in gene family size is concerned.

## Methods

### *E. coli *regulatory network

Regulatory interactions in the *E. coli K12 *transcriptional network and operon structures were obtained from RegulonDB 6.2 [13,39]. TFs were grouped into four regulatory types: those with "no auto-regulation" (N = 70), "negative auto-regulation" (N = 64), "positive auto-regulation" (N = 26), and "dual auto-regulation" (N = 9, where both positive and negative auto-regulation have been reported) based on their annotation in RegulonDB. Groupings are mutually exclusive. TFs that were not explicitly called "dual" in RegulonDB but annotated with both auto-repressive and auto-activating interactions (N = 2) in the same resource were assigned to the "dual" class.

### *E. coli *gene duplications

Sequence information for all protein-coding genes in the *E. coli K12 *genome was extracted from genomic sequence downloaded via NCBI Entrez (NC_000913) using custom Tcl/Tk scripts. This information was used to compute gene lengths (see "Regulatory types do not differ in gene complexity").

Homologous relationships between genes are typically inferred using primary (nucleotide or protein) sequence information. However, as sequences diverge from each other over time, one might fail to detect homology between anciently diverged paralogs and falsely assign these genes to unrelated gene families. As the structure of a protein is more conserved than its sequence, assigning gene family membership based on structural homology affords greater resolution of homologous relationships for older duplicates. In addition, defining gene families based on features of demonstrable functionality (domains) should provide a more functionally cohesive grouping compared to assigning genes to families based on often rather arbitrary sequence similarity cut-offs. As one might expect, gene family partitions are more inclusive than those based on primary sequence, and hence more conservative in identifying single-gene families [40]. We therefore adopted an approach taken by Teichmann and Babu [40] to detect homologous relationships amongst *E. coli *genes. For each protein-coding gene in the *E. coli *genome, we screened the SUPERFAMILY database [41,42] of protein domains for significant domain hits. Following Teichmann and Babu we refer to the ordered array of domains, from the amino to the carboxyl terminus of the polypeptide chain, as the domain architecture of the protein. Genes that share the same domain architecture (ignoring domain repeats and gaps) we considered to be derived from a common ancestor and hence members of the same gene family, i.e. duplicates. Conversely, genes with a unique domain architecture were considered to have no recognizable paralogs and thus to constitute single-gene families. The SUPERFAMILY database is fundamentally based on domain classifications from the SCOP database, which classifies domains at three levels of hierarchy: family, superfamily, and fold. Domains that belong to the same family share clear sequence similarities. The superfamily is more inclusive and can thus contain domains where sequence similarity is reduced but where there is still structural or functional evidence for common evolutionary descent. Finally, superfamilies clustered together at the fold level share the same broad-scale secondary structure and chain topology but no clear evolutionary relationship is evident. We thus assigned domains at both the family and superfamily level. Results are qualitatively identical and we therefore only present data for the "family" level. As the absence of a recognizable domain does not by itself imply that the gene in question has no paralogs, we restricted our analysis to genes with at least one significant domain hit (>90% of TFs), leaving 9, 62, 24, and 60 TFs with dual, negative, positive, and without auto-regulation, respectively (see Table 1 and Additional file 1). Treating genes without domain hits as singletons does not alter our conclusions. Note that for the purpose of this study identification of duplicate relationships beyond the family level is not required as we differentiate only between genes in single-gene families and those in multi-gene families.

### Potential confounding factors in *E. coli*

Data on protein under-wrapping was taken from Supplementary Table One of Liang *et al*. [27]. As computation of under-wrapping requires information on the structure of the protein concerned, we could assign under-wrapping coefficients only to a subset of TFs (N = 47).

Data on horizontal gene transfers was taken from [43]. Briefly, this data reflects the most parsimonious scenario of gene gains and losses across the tree of 21 proteobacteria, with relative penalties for gains and losses of 2:1 and using the DELTRAN algorithm implemented in PAUP*.

### *S. cerevisiae *transcription network

The transcription network of *S. cerevisiae *was obtained from refs. [22,44]. Genes in the network were classified into the same four categories as for the *E. coli *network. There are only a small number of TFs that exhibit positive (N = 10) and negative (N = 4) auto-regulation. The 'dual auto-regulation' class for yeast is empty and the remaining 112 TFs show no evidence for auto-regulation.

### *S. cerevisiae *gene duplications

Sequence information for *S. cerevisiae *was taken from [45]. In contrast to *E. coli*, a substantial proportion (~40%) of TFs in this study curiously do not have a significant domain hit in the SUPERFAMILY database. As discussed above, absence of a recognizable domain does not by itself imply that the gene in question has no paralogs. Eliminating these cases, as done for *E. coli*, however, would have severely reduced an already small sample. We thus decided to define gene families in *S. cerevisiae *based on sequence similarity. We identified gene pairs with similar sequences using an all-against-all blast analysis of *S. cerevisiae *proteins. Protein pairs with blast e-value < 10^-10 ^and an alignment length of at least 150 amino acids were regarded as duplicates.

It is not the case that, in the context of this analysis, homology inference by sequence comparison is adequate in yeast but not *E. coli*. We persist with the structural homology method for *E. coli *because we consider it superior and, in contrast to yeast, the widespread availability of significant domain hits allows us to go down that route without losing an appreciable amount of data.

For a list of all TFs analyzed in this study for both *E. coli *and *S. cerevisiae *alongside relevant characteristics see Additional file 1.

## Authors' contributions

TW and MJL conceived of and coordinated the study and drafted the manuscript. TW compiled and processed the *E. coli *data. GZW compiled and processed the *S. cerevisiae *data and helped draft the manuscript. LDH participated in the design and coordination of the study and helped draft the manuscript. All authors participated in the data analysis. All authors read and approved the final manuscript.

## Supplementary Material

Additional file 1 **Family assignment and other relevant characteristics of transcription factors used in this study**. List of *E. coli *and *S. cerevisiae *transcription factors analyzed in this study alongside information on domain architecture, auto-regulatory interactions, gene family size and other relevant characteristics. Click here for file
